# Transcriptional Repression of p53 by PAX3 Contributes to Gliomagenesis and Differentiation of Glioma Stem Cells

**DOI:** 10.3389/fnmol.2018.00187

**Published:** 2018-06-08

**Authors:** Hui Zhu, Hongkui Wang, Qingfeng Huang, Qianqian Liu, Yibing Guo, Jingjing Lu, Xiaohong Li, Chengbin Xue, Qianqian Han

**Affiliations:** ^1^Jiangsu Clinical Medicine Center of Tissue Engineering and Nerve Injury Repair, Research Center of Clinical Medicine, Affiliated Hospital of Nantong University, Nantong, China; ^2^Key Laboratory of Neuroregeneration of Jiangsu and Ministry of Education, Co-innovation Center of Neuroregeneration, Nantong University, Nantong, China; ^3^Department of Neurosurgery, Affiliated Hospital of Nantong University, Nantong, China; ^4^National Institute for Food and Drug Control, Beijing, China

**Keywords:** PAX3, p53, glioma, stem cells, tumor microenvironment

## Abstract

Although there are available therapies as surgery, chemotherapy and radiation, glioblastoma (GBM) still has been considered as the most common and overwhelming primary tumor of brain. In GBM, the brain glioma stem cells (BGSCs) were identified and played a crucial role in resistance of GBM to conventional therapies described above. PAX3 was previously identified by our group as a diagnostic/prognostic marker and a therapeutic regulator in the therapy of GBM. Here, we hypothesized PAX3/p53 axis promoted the process of differentiation, regulating to the cancer stem cell properties, such as proliferation and migration. The correlation between PAX3 and p53 in GBM were first clarified. Immunofluorescence of p53 was shown activated following BGSCs differentiation. We further identified that PAX3 might specifically bind to the promoter of p53 gene, and transcriptionally repressed p53 expression. ChIP assay further confirmed that PAX3/p53 axis regulated the differentiation process of BGSCs. Then, the function of PAX3 in BGSCs were sequentially investigated* in vitro* and* in vivo*. Ectopic PAX3 expression promoted BGSCs growth and migration while PAX3 knockdown suppressed BGSCs growth, migration *in vitro* and *in vivo*. Similar to PAX3 overexpression, p53 inhibition also showed increase in growth and migration of differentiated BGSCs. Regarding the functional interaction between PAX3 and p53, PAX3 knockdown-mediated decrease in proliferation was partially rescued by p53 inhibition. Hypoxia significantly promoted the migration potential of BGSCs. In addition, hypoxia inducible factor-1α (HIF-1α) might be a potential upstream regulator of PAX3 in differentiated BGSCs under hypoxia. Our work may provide a supplementary mechanism in regulation of the BGSCs differentiation and their functions, which should provide novel therapeutic targets for GBM in future.

## Introduction

Brain tumors are generally classified according to pathological features. Among them, non-malignant corresponds to Grade I and II tumors, and malignant corresponds to Grade III and IV tumors, while glioblastoma (GBM) corresponds to Grade IV tumors (Louis et al., 2016). As a most common/malignant brain tumor, GBM provides a limited life expectancy that can be measured in weeks rather than years by traditional therapies, including radiation and chemotherapy. Therefore, there is a pressing need to further investigate GBM biology and push the translation of related research results to therapy (Ludwig and Kornblum, 2017).

Mainly owing to the invasive potential, incomplete resection and increased resistance to treatment, malignant glioma owns the poor prognosis. (Lee et al., 2015). The recurrence of GBM has been well known to be resistant to radiation and chemotherapy that caused by brain glioma stem cells (BGSCs; Beier et al., 2011). Originally discovered in GBM, BGSCs likely play crucial roles in resistance of tumors to conventional therapies and vascular formation of glioma (Liebelt et al., 2016). Besides microenvironmental factors, these features present potential targets for precisely targeting therapy against the BGSCs in GBM (Liebelt et al., 2016). Therefore, BGSCs have been considered as a relevant target for GBM therapy, and the elimination of BGSCs is crucial in treating GBM. The strategy to target BGSCs therapeutically is mainly focused on the direct ablation of BGSCs by targeting cell surface markers and specific pathways that are required for maintaining BGSCs stemness. However, it has been increasingly acknowledged that another way to specifically target BGSCs is to alter the ability of BGSCs to interact with their microenvironment.

Compared to neural stem cells (NSCs), BGSCs exhibit enhanced self-renewal capacity and compromised differentiation (Hu et al., 2013). BGSCs upregulate a number of signaling pathways required for maintaining NSC stemness, which enables them to enhance their stemness and aberrant cell survival, consequently leading to tumorigenesis (Hemmati et al., 2003; Vescovi et al., 2006; Rich and Eyler, 2008). Accordingly, research on the BGSCs has become a high significance for therapeutic progress. It is pressing to clarify the biological and molecular mechanisms of GBM progression, founded on which to develop additional operative therapies (Tang et al., 2016).

PAX3 is a member of PAX gene family. It has been reported to involve in oncogenesis (Wang et al., 2008), which maybe upregulated in all GBMs, melanomas, gastric cancers, Ewing sarcomas, rhabdomyosarcomas and neuroblastomas (Schulte et al., 1997; Frascella et al., 1998; Plummer et al., 2008; Xia et al., 2013; Fang et al., 2014; Zhang et al., 2015). These works suggest that PAX3 may be a potential oncogene in tumorigenesis. Abnormal expression of PAX genes in any adult tissue may be highly correlated with various tumors induced by sponsoring or impeding tumorigenesis (Robson et al., 2006; Li and Eccles, 2012). Pax3 acts in a p53-dependent way to inhibit cell apoptosis, however, the proliferation of brainstem progenitors may be promoted by Pax3 without p53 (Misuraca et al., 2014). Pax3 is not necessary to function as a transcriptional regulator during closure of neural tube and development of cardiac neural crest, however, inhibiting p53 is the unique requirement exerting Pax3 function (Wang et al., 2011).

Our group previously reported that the expression of PAX3 was upregulated in tissue specimens of GBM with high-grade rather than low-grade or normal. The PAX3 is highly expressed with the increase in tumor WHO grade (Chen et al., 2012). PAX3 is also a crucial factor in both differentiation of NSCs into astrocytes and the regulation of biological properties of GBM cells. Meanwhile, as both diagnostic/prognostic marker and novel therapeutic target in the therapy of GBM, PAX3 supposed to be an intrinsic regulator in development of GBM (Xia et al., 2013).

Our present work provides a set of novel clinical and molecular mechanistic findings that hypoxia inducible factor-1α (HIF-1α) mediated PAX3/p53 axis is an essential mechanism that controls self-renewal, migration, and tumorigenesis of BGSCs in a p53-dependent manner. Our work may provide a supplementary mechanism in regulation of the BGSCs differentiation and their functions, which should provide novel therapeutic targets for GBM in future.

## Materials and Methods

### Tissue Microarray and Immunohistochemistry

Tissue microarrays were operated by Shanghai Zhuoli Biotechnology Co., Ltd in China. The array contained six normal brain tissues, 36 grade I–II, 47 grade III–IV tumor samples. To carry out ultraviolet cross linkage, each sample from paraffin tissue blocks were cut into the 1–2 μm thick sections, dewaxed, pretreated and transferred to the glass slides by an adhesive tape transfer system. All the steps described above were accomplished using an automated staining equipment. Two independent researchers who were blinded to patient details completed the quantitation of immunostaining for PAX3 and mutant p53. According to immunostaining intensity and distribution, the immunostaining score was divided into four grades: − (score 0), + (score 1), ++ (score 2) and +++ (score 3). The positive rates were scored as 0 point (<5%), 1 point (5%–30%), 2 points (30%–70%), 3 points (>70%). The final immunostaining score was calculated based on the sum of intensity, distribution and positive rates, which defined as follows: no expression (total score 0), weak expression (total score 1 and 2), moderate expression (total score 3 and 4) and strong expression (total score 5 and 6; Ma et al., 2016; Shen et al., 2016).

### Cell Culture and Reagents

Human BGSCs established from high-grade gliomas were kindly gifted by Soochow University, China. In the state of neuro-spheres, BGSCs grew in a stem cell-permissive medium comprising of Dulbecco’s modified Eagle’s medium (DMEM)-F12 supplemented with B27 (1:50; Life Technologies, Carlsbad, CA, USA), adding basic fibroblast growth factor (20 ng/ml) and epidermal growth factor (EGF; 20 ng/ml). P3 BGSCs were used for the study. The BGSCs were cultured in DMEM-F12 medium containing 10% FBS for 7 days to induce differentiation (Sareddy et al., 2017). Purchased from the American Type Culture Collection (ATCC, MD), Human HEK-293T, U251 MG and U87 MG cells were cultured in DMEM supplemented with 10% FBS and Penicillin, Streptomycin in 100 mm dishes in a humidified atmosphere containing 5% CO_2_ at 37°C. The normal astrocytes (1800) were cultured in modified RPMI-1640 medium supplemented with 10% FBS and Penicillin. The CoCl_2_•6H_2_O (Sigma-Aldrich) was used to simulate hypoxia. The hypoxia control group was treated with 500 μmol/L CoCl_2_ for 48 h, while the normoxia control group was solely treated with an equal volume of the solvent (DMSO; Gopisetty et al., 2013; Liang et al., 2014).

### Sphere Formation Assay

For sphere formation assays, cells with a density of 5 × 10^3^ cells/ml were dissociated and suspended in stem cell culture medium; then the cell suspension was divided into a non-coated 96-well plate, 100 μl for each well. The quantity of tumor spheres formation on 96-well plates was accomplished 3 days post-seeding under phase-contrast microscopy (Sato et al., 2013).

### Immunofluorescence Staining

The cell coverslips were fixed with 4% paraformaldehyde for 20 min at room temperature. After washing twice (10 min for each time) with PBS, they were permeabilized with 0.1% Triton X-100/PBS for 10 min at room temperature, and then twice washing (10 min for each time) with PBS. Sequentially, the cell coverslips were incubated with blocking solution for 30 min. We carried out the incubation with anti-PAX3 (1:500, Abcam), anti-p53 (1:200, Cell Signaling Technology), anti-Nestin (1:200, Millipore), anti-CD15 (1:100, Proteintech), anti-CD133 (1:100, Cell Signaling Technology) or Cleaved Caspase-3 (1:100, Cell Signaling Technology) for at least 2 h at room temperature. Followed by observation under a fluorescence microscope, the cell coverslips were washed three times (10 min for each time) with PBS and fluorescence Cy3 or FITC-labeled secondary antibodies were added for additional incubation (1 h at room temperature; Lu et al., 2013).

### RNA Extraction and Quantitative Real-Time PCR

According to the manufacturer’s instructions, the TRIzol^®^ reagent (Invitrogen) was used to isolate the total RNA of cells and tissues. By the aid of ABI 7500 Real-Time PCR System (Applied Biosystems, Waltham, MA, USA), quantitative real-time polymerase chain reaction (qRT-PCR) was carried out in triplicate and normalized with expression of GAPDH. The relative expression level was calculated using the comparative 2^−ΔΔ^Ct method (Zhang et al., 2014). The primers used were as follows (5′-3′): PAX3, GCTGGGAAATCCGAGACA (forward) and CCTCCTCCTCTTCACCTTT (reverse); p53, AGCTTTGAGGTGCGTGTTTGTG (forward) and TCTCC ATCCAGTGGTTTCTTCTTTG (reverse); HIF-1α, GTGTAC CCTAACTAGCCG (forward) and CAAATCAGCACCAAGC (reverse).

### Western Blot Analysis

Extracted from cell cultures, the protein samples were separated by SDS-PAGE and transferred to the PVDF membranes. According to the manufacturer’s recommendations, the PVDF membranes were then blocked and incubated with the primary antibodies against PAX3 (1:500, Abcam), p53 (1:200, Cell Signaling Technology), Cleaved Caspase-3 (1:500, Cell Signaling Technology), Bcl-2 (1:500, Proteintech) or GAPDH (1:1000, Proteintech). Via HRP-conjugated species-specific secondary antibody (Beyotime, Shanghai, China) and enhanced chemiluminescence assay, the specific binding of primary antibody was finally detected (Zhang et al., 2016).

### Transient Transfections Gene Silencing by siRNA

siRNAs against human PAX3, p53, HIF-1α and non-targeting control were purchased from Ribobio (Guangzhou, China). According to the manufacturer’s instructions, transfection of siRNAs was executed by Lipofectamine RNAi MAX (Invitrogen; Sato et al., 2013).

### Lentivirus and Plasmid Transfection

The recombinant lentivirus for PAX3-EGFP (Lv-PAX3) and PAX3 knockdown lentivirus (Lv-shPAX3) were purchased from Genechem (Shanghai, China). PAX3 knockdown by shRNA lentivirus with the most effective sequence (5′-3′): CCGCATCCTGAGAAGTAAA. PAX3 overexpression plasmid was constructed by GeneChem (Shanghai, China). According to the manufacturer’s instructions, transient transfections in HEK-293T cells were carried out by Lipofectamine RNAiMAX transfection reagent (Invitrogen).

### Cell Viability Assay

According to the manufacturer’s instructions, the cell proliferation was determined by a Cell Counting Kit-8 (CCK-8, Dojindo, Japan). Briefly, after transfection with Lv-shPAX3 or Lv-PAX3, BGSCs were seeded onto 96-well plates (5000 cells in 100 μl of stem cell medium/well). Forty-eight hours later, 10 μl of CCK-8 solution was added after the substitution with 100 μl fresh DMEM media (Gibco, Carlsbad, CA, USA). Then, the whole plates were incubated at 37°C for 2 h. By the aid of a microtiter plate reader (BIO-TEK, Winooski, VT, USA), the absorbance of each well was measured at 450 nm, while the viability of scrambled control-transfected BGSCs was assumed to be 100% (Zhang et al., 2014).

### Transwell Assay

After detaching and suspending in serum-free medium, 3 × 10^4^ cells for each well were plated on the top chamber of transwells (24-well insert; pore size, 8 μm). Toward DMEM added with 10% FBS, the cells were permitted to migrate into the lower chamber. In the upper chamber, non-migrating cells were removed from the upper surface of the filters using a PBS-soaked cotton swab after the incubation for 20 h. Followed by fixation using 4% paraformaldehyde and stained using 0.1% crystal violet, the quantity of migrating cells was completed by counting three different fields under a phase-contrast microscope. To investigate the migration of BGSCs under hypoxic conditions, cells were cultured in 1% O_2_/5% CO_2_/ balance N_2_ in a modulator incubator chamber at 37°C.

### Tumorigenicity Assay

All experimental protocols were approved by the Administration Committee of Experimental Animals, Jiangsu Province (SYXK (Su) 2012-0031), China, which is in accordance with the US National Institute of Health (NIH) Guide for the Care and Use of Laboratory Animals published by the US National Academy of Sciences. The present study obtained ethical approval from the Affiliated Hospital of Nantong University, China (approval ID: 2014-021). For tumorigenicity assays, near the upper extremity of 16 Balb/C female nude mice (6 weeks old) were injected with the stably transfected cells or control cells (2 × 10^6^) subcutaneously into the right front. We monitored the nude mice weekly and calculated tumor volume (mm^3^) by a standard formula: length × width × height × 0.5236. Eight weeks after implantation, all nude mice were sacrificed and tumors were harvested and weighed individually before the fixation. Data were showed as tumor volume (mean ± SE) and tumor weight (mean ± SE; Xia et al., 2013). Then tumors were processed for histological studies. Animal experiments were performed in strict accordance with the Institutional Animal Care guidelines.

### Luciferase Reporter Assay

The promoter region of the human p53 gene was analyzed by Genomatix software to identify potential binding sites for transcription factors. The potential PAX3 protein binding site (5’-cttgtCATGgcgactgtcc-3′, spanning from −222 bp to −203 bp on the positive strand), was identified by the matrices database of transcription factor binding sites. We cloned a two kilo-base region upstream of the transcription start site of p53 into the GV354 vector upstream of the luciferase gene in order to determine whether PAX3 regulates the promoter activity of p53. By the aid of oligonucleotide directed deletions with Pfu Turbo DNA polymerase from a QuikChange kit (Stratagene), we then constructed mutated p53 promoter by specifically delete PAX3 binding elements. Using a Dual-Luciferase Reporter Assay System (Promega) on the BioTek Synergy 2, the promoter activity was detected and Renilla luciferase activity was used as an internal control. After normalization relative to the Renilla luciferase activity, the firefly luciferase activity was calculated as the mean ± SE (Tang et al., 2016).

### Electrophoretic Mobility Shift Assay

Electrophoreticmobility shift assays (EMSAs) were performed after preparing nuclear extracts of HEK293T cells transfected with Lv-PAX3. A synthetic oligonucleotide covering the region of the human p53 promoter labeled with biotin was obtained from Sangon Biotech Co. Ltd. (Shanghai, China). In this experiment, the sequences of normal and mutated oligonucleotides were as follows (5′-3′): normal, CTTGTCATGGCGACTGTCC (forward) and GGACAGTCGCCATGACAAG (reverse); mutated, CTTGGACGTGCGACTGTCC (forward) and GGACAGTCGCACGTCCAAG (reverse; mutation within the core Pax3 binding domain).

### Chromatin Immunoprecipitation

Following the manufacturer’s instructions, the ChIP assay was performed by the EZ-CHIP™ chromatin immunoprecipitation kit (Merck Millipore). Briefly, BGSCs and differentiated glioma cells were lysed, and anti-PAX3 polyclonal antibodies (sc-34918, Santa Cruz Biotechnology) was used in the chromatin immunoprecipitation. In ChIP-qPCR assay, primers of the p53 promoter were as follows (5′-3′): ATGTTAGTATCTACGGCACCAG (forward) and CAGCCCGAACGCAAAGTG (reverse). By ABI 7500 with SYBR Green detection (Applied Biosystems), qRT-PCR was carried out according to the standard protocol. To account for chromatin sample preparation differences (ΔCp_Normalized ChIP_), input DNA (non-IP enriched) values were used to normalize each ChIP DNA fraction’s Cp (crossing point) value inputting DNA fraction Cp value. Based on the normalized IgG only IP fraction Cp value ΔΔCp = (ΔCp_Normalized ChIP_ − (ΔCp_Normalized IgG_)), the normalized ChIP fraction Cp values were adjusted. Above the sample specific background, the ChIP assay site fold enrichment was then calculated as 2^(−ΔΔCp)^ (Brooks et al., 2016).

### Statistical Analysis

In tissue microarray, Chi-square test was applied to compare the data, and Fisher’s exact test was used to test for significance of the association between high PAX3 expression and p53 mutations. The data are expressed as the mean ± SEM. The data were replicated in three experiments at least of statistical analyses. Either a two-tailed Student’s *t*-test or one-way ANOVA followed by Bonferroni *post hoc* *t*-test was used to calculate statistical significance between two groups. Significant differences were considered as *p* < 0.05 (*) and *p* < 0.01 (**).

## Results

### High Expression of PAX3 and Mutant p53 in Human Glioma Tissue

**Figure 1:**
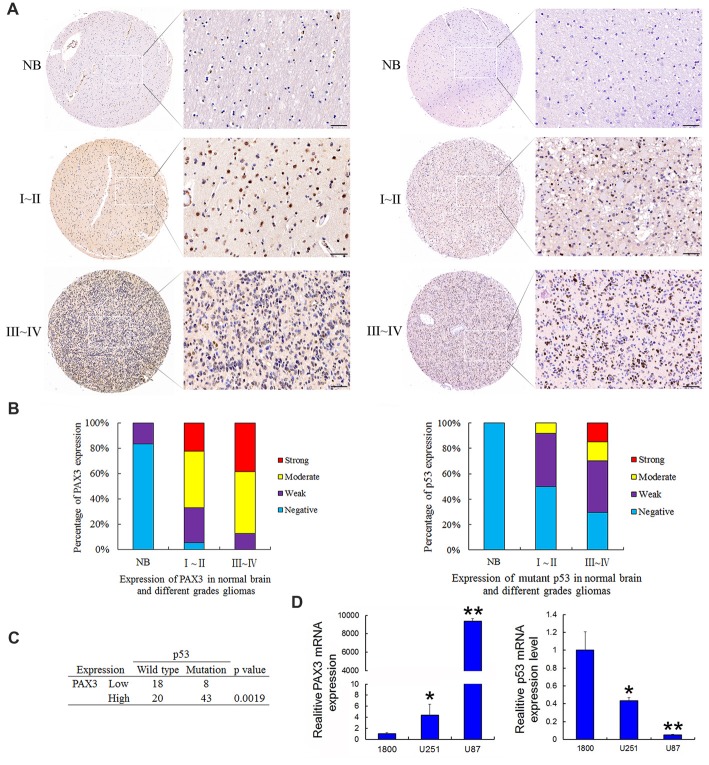
Expression of PAX3 and mutant p53 in the human glioma tissue microarray. **(A)** Representative sections for PAX3 and mutant p53 immunoreactivity in normal brain and different grades of gliomas (400×). **(B)** Overall level of PAX3 and mutant p53 expression was significantly than in WHO III–IV gliomas tissues compared with WHO I–II gliomas tissues and normal brain tissues according to the result of immunohistochemistry (IHC; *p* < 0.01). **(C)** Table showing positive correlation between PAX3 expression level and p53 mutations from the dataset in **(A)**. **(D)** Expression of PAX3 and p53 mRNA in human glioma cell lines (U87, U251) and the normal astrocytes 1800. *p < 0.05 and **p < 0.01 vs. 1800.

Tissue microarrays were performed by Shanghai Zhuoli Biotechnology Co., Limited (Zhuoli Biotechnology Co., Shanghai, China). The array contained six normal brain tissues, 36 grade I–II, 47 grade III–IV tumors. Both PAX3 and mutant p53 was immuno-stained at the nucleus. As shown in Figure 1A, the intensity of both PAX3 and mutant p53 immunostaining in cancer tissue were significantly stronger than in normal brain tissues, corresponding to the tumor grade. In glioma specimens, 97.6% showed a positive PAX3 expression, while it was only 16% in normal brain tissues. Furthermore, the occurrence of PAX3 immuno-reactive specimens with moderate or strong PAX3 expression in grade I–II to III–IV was 66.6% and 87.2% (Figure 1B). The relative expression level of PAX3 closely correlated with the pathological grades (*p* < 0.01). These results confirmed our previous findings that PAX3 is associated with the early stage of glioma-genesis (Chen et al., 2012).

It has been previously reported that p53 is a well-known molecular marker in glioma (Ludwig and Kornblum, 2017). As a moderately sensitive and highly specific marker, p53 immuno-positivity has been used to predict p53 mutations in GBM (Takami et al., 2015). The occurrence of p53 immuno-reactive specimens with moderate or strong mutant p53 expression in grade I–II to III–IV was 50% and 70.2%. These results suggested p53 inactivation also associated with glioma-genesis. All these results illustrated that the increased PAX3 expression, as well as p53 inactivation in glioma tissues, is correlated with gliomas progressing from low to high grades. We next compared p53 mutations occurring in PAX3-Low vs. PAX3-High glioma samples. As is shown in Figure 1C, more p53 mutations occur in PAX3-High tumors, suggesting a link between PAX3 and p53 expression. Furthermore, we examined the expression of PAX3 in the established human glioma cell lines (U87 MG, U251 MG) and the normal astroglial cell line, 1800. Results showed that PAX3 mRNA was highly expressed in glioma cells compared with normal astrocytes, while the expression of p53 mRNA was low in glioma cells (Figure 1D).

### PAX3 Is Highly Expressed in BGSCs While Decreased After Differentiation

**Figure 2:**
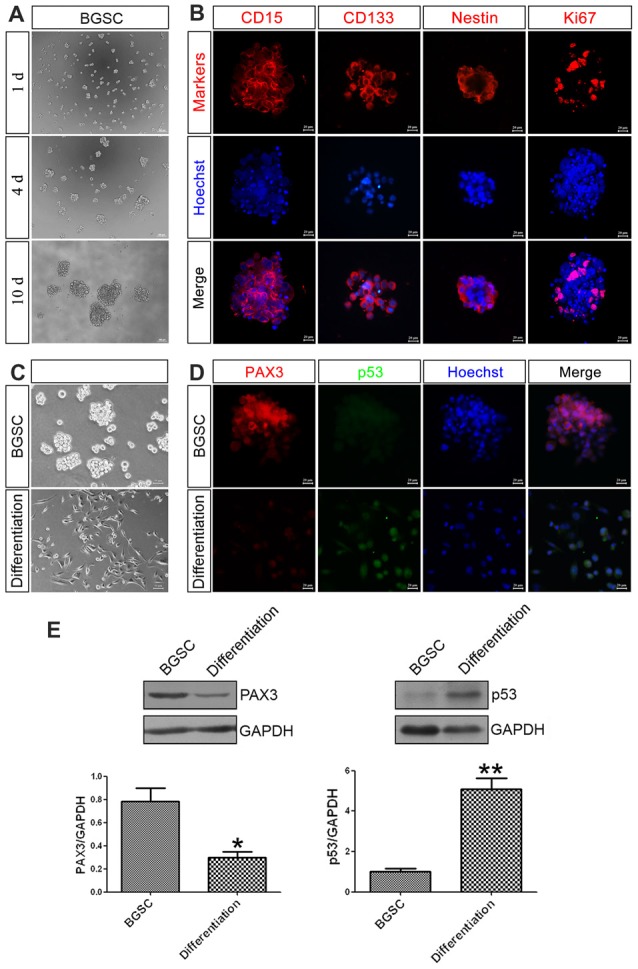
PAX3 is highly expressed in BGSCs while decreased after differentiation. **(A)** Brain glioma stem cells (BGSCs) could form neurospheres from single cells by proliferation. **(B)** BGSCs expressed neural stem cell marker (CD15, CD133, nestin) and proliferation marker Ki67. **(C)** BGSCs were cultured in stem cell culture medium or differentiation medium containing 10% FBS for 7 days. **(D)** Then they were subjected to immunofluorescence analysis for PAX3 (red) and p53 (green). Nuclei were stained with Hoechst 33342 (blue); Scale bars: **(A)**, 100 μm; **(B,D)**, 20 μm; **(C)** 50 μm). **(E)** Changes of PAX3 and p53 protein expression level in BGSCs and differentiation by western blot analysis. *p < 0.05 and **p < 0.01 vs. BGSCs.

As a key component in GBM initiation, invasion and recurrence, BGSCs were identified by immunofluorescence analysis with three markers of BGSCs, namely CD15, CD133 and nestin. The results showed that the neurosphere-like spheroids formed from single cells by proliferation and were positive for the stem cell markers (Figures 2A,B). BGSCs were induced differentiation by culturing these spheroids under differentiating conditions (Figure 2C). The expression of both genes in BGSCs and differentiated BGSCs were determined to investigate the biological functions of PAX3 and p53 in regulation of BGSCs. Results showed that PAX3 as a stemness gene was highly expressed in BGSCs and then downregulated. p53 is an important determinant factor of glioma stem cell differentiation (Zheng et al., 2008; Gu et al., 2013). Thus, we explored the potential roles of PAX3 in BGSCs. Immunofluorescence staining and western blot analysis were applied to detect the expression of PAX3 and p53 for exploring the change of PAX3 and p53 during BGSCs differentiation. Results showed that PAX3 was highly expressed in BGSCs while decreased after differentiation. Reversely, p53 activation was detected after BGSCs differentiation, suggesting they had an opposite expression pattern (Figures 2D,E).

### PAX3 Knockdown Suppresses BGSCs Growth, Migration *in Vitro* and *in Vivo*

**Figure 3:**
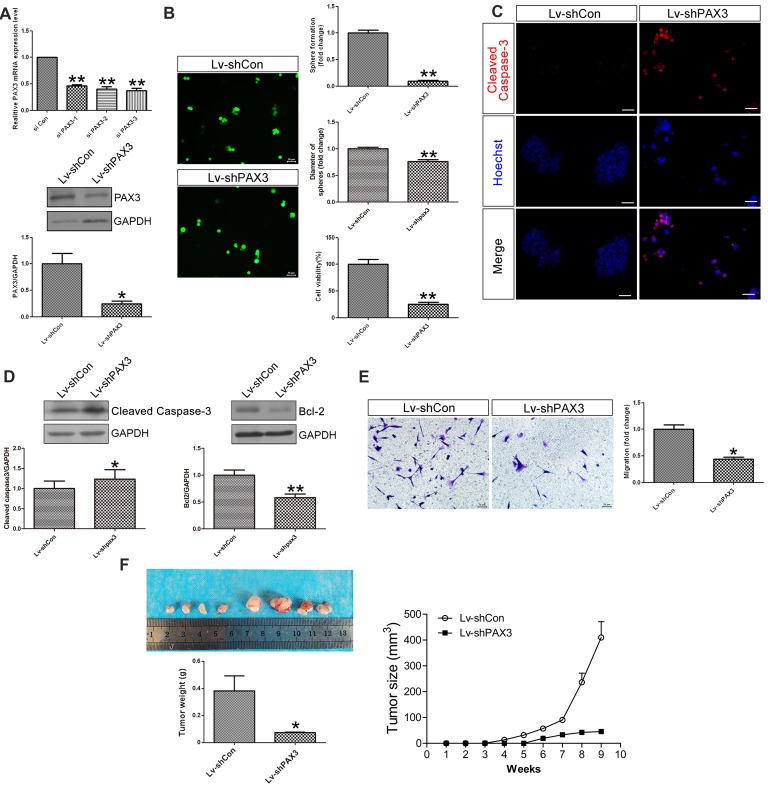
PAX3 knockdown suppresses BGSCs growth, migration *in vitro* and *in vivo*. **(A)** Quantitative real-time polymerase chain reaction (qRT-PCR) and western blot analysis showed that the mRNA and protein expression of PAX3 were significantly decreased by knockdown with siRNA-3. **(B)** PAX3 knockdown reduced the colony formation and size of BGSCs significantly after 3 days by sphere formation assay. PAX3 knockdown also decreased the proliferation of BGSCs, as determined by a CCK-8 assay. **(C)** Cleaved Caspase-3 immunostaining for BGSCs showed an increase in cell apoptosis by PAX3 knockdown. **(D)** Western blot analysis was performed to determine the expression of apoptosis-related genes (Cleaved Caspase-3 and Bcl2). **(E)** Representative images and quantification of transwell migration assay indicated that downregulation of PAX3 inhibited the BGSCs migration. **(F)** Tumor growth curves and tumor weight of mice with xenografts locally injected with lentivirus transfected BGSCs. Scale bars: **(B,C,E)**, 50 μm. *p < 0.05 and **p < 0.01 vs. negative control (Lv-shCon).

To explore the biological functions of PAX3 in BGSCs, we knocked down the PAX3 expression by siRNA. By the aid of siRNA-3 and shRNA lentivirus, qRT-PCR and western blot analysis showed that both mRNA and protein expression of PAX3 were significantly decreased (Figure 3A). As one of the most important characteristics of cancer stem cells, the self-renewal ability of BGSCs can be detected by sphere formation assay. Results showed that knocking down of PAX3 significantly inhibited sphere-forming capacity and cell proliferation (Figure 3B) as compared with controls. We also detected the apoptosis-related genes (Cleaved Caspase-3 and Bcl2) by immunofluorescence analysis and western blot analysis (Figures 3C,D). Results indicate that the decrease in BGSCs growth is due to proliferation defects and an increase in cell apoptosis. In addition, transwell migration assay indicated that downregulation of PAX3 significantly inhibited the BGSCs migration (Figure 3E). Furthermore, the effect of PAX3 on BGSCs *in vivo* was confirmed by tumorigenicity assay. Results showed that significant decrease in the tumor weight and the tumor size of the BGSCs transfected by shPAX3 lentivirus at 9 weeks after injection (Figure 3F).

### PAX3 Transcriptionally Represses p53 Expression in BGSCs and Differentiation

**Figure 4:**
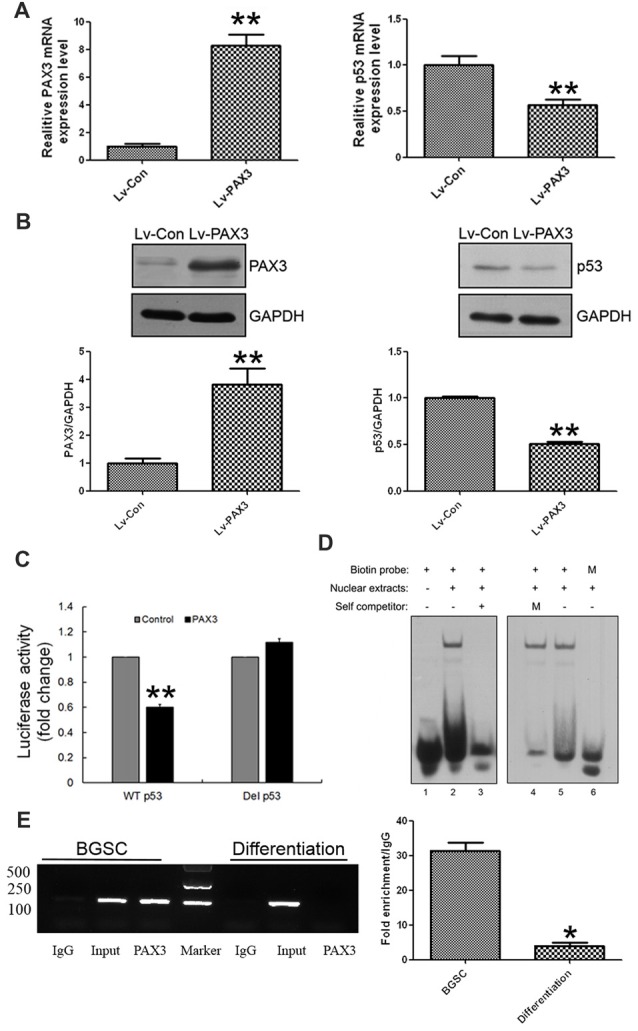
PAX3 transcriptionally represses p53 expression in BGSCs and differentiation. p53 expression in mRNA **(A)** and protein **(B)** levels were detected by qRT-PCR and western blot after PAX3 overexpression. **(C)** Luciferase reporter assay was used for the luciferase activity driven by p53 promoter in HEK293T cells after PAX3 overexpression. **p < 0.01 vs. control. **(D)** Nuclear extracts from HEK293T cells ectopically expressing PAX3 were incubated with biotin labeled probes to determine whether they could form the complex. Lane 1, negative control with probe only. Lane 2 and 5, nuclear extracts from PAX3 ectopically expressed HEK293T cells. Lane 3, nuclear extracts were incubated with 200-fold molar excess unlabeled oligonucleotide of identical probe sequence in addition (self-competitor). Lane 4, nuclear extracts were incubated with mutated 200-fold molar excess unlabeled oligonucleotide. Lane 6, nuclear extracts were incubated with mutated biotin labeled probes. **(E)** Chromatin immunoprecipitation assays indicating that PAX3 occupied the promoter of the endogenous p53 gene in BGSCs, but not in differentiated BGSCs. ChIP-qPCR was used detect the enrichment of binding sites. *p < 0.05, **p < 0.01 vs. control (Lv-Con).

Considering that PAX3 expression was inversely correlated with p53 expression during BGSCs differentiation, we examined the role of PAX3 in regulating p53 expression. After PAX3 overexpression, p53 expression was markedly reduced in differentiated BGSCs of both qRT-PCR analysis (Figure 4A) and western blot (Figure 4B). The results suggest that PAX3 is an upstream regulator of p53. We cloned p53 promoter into the GV354 reporter plasmid to confirm the effect of PAX3 on the transcriptional activation of p53 promoter. Luciferase assay results showed that over-expression of PAX3 significantly reduced the transcription activity of wild type p53 promoter (WT p53), while absence of PAX3 binding site in the promoter (Del p53) failed to affect the transcription activity (Figure 4C). The PAX3 binding sequences are negative regulatory elements for p53 transcription, which was demonstrated by our results. Then, with the nuclear extracts of PAX3-expressing HEK293T cells, the double-stranded oligonucleotides corresponding to the predicted cis-element in the p53 promoter formed sequence-specific DNA-protein complexes demonstrated by electrophoretic mobility shift assay (EMSA). However, such a DNA-protein complex failed to be generated by the mutated probes in the EMSA (Figure 4D). In the putative p53 promoter of BGSCs, PAX3 protein was exactly recruited to the two binding sites, and their differentiation resulted into decrease of the binding level, which further confirmed by chromatin immunoprecipitation qPCR (ChIP-qPCR) assay (Figure 4E). These data suggest that PAX3 transcriptionally represses p53 expression and PAX3/p53 axis regulates the BGSCs differentiation process.

### PAX3 Overexpression Promotes BGSCs Growth and Migration

**Figure 5:**
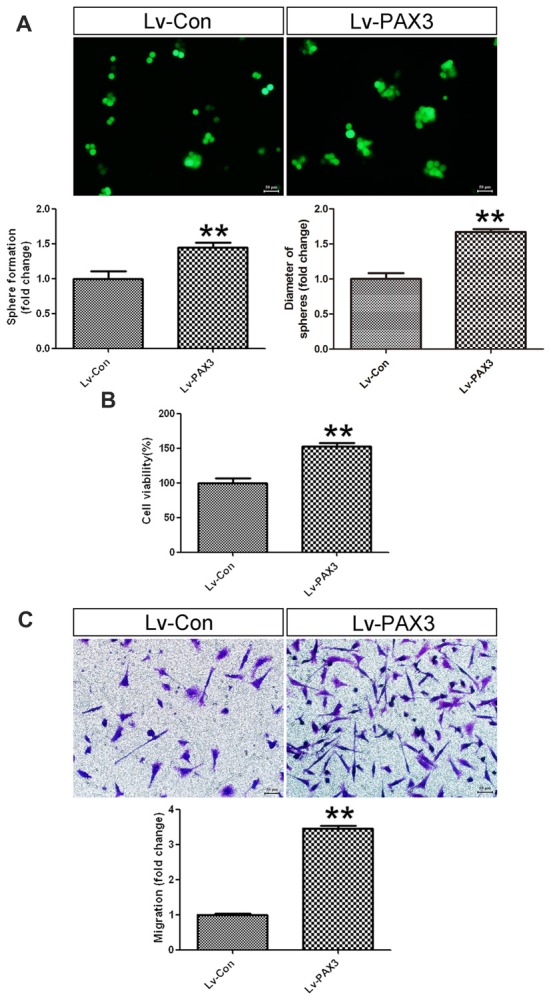
PAX3 overexpression promotes BGSCs growth and migration. **(A)** Sphere formation assay showed that PAX3 increased the colony formation and size of BGSCs. Overexpression of PAX3 increased the proliferation **(B)** and migration **(C)** of BGSCs. **p < 0.01 vs. control (Lv-shCon).

To clarify the effects of PAX3 overexpression on sphere-forming capacity, cell viability and migration were investigated subsequently. As shown in Figure 5A, BGSCs transfected with Lv-PAX3 increased the sphere formation and colony size as compared with control (Figure 5A). In addition, ectopic expression of PAX3 significantly increased cell viability (Figure 5B) and migration (Figure 5C) compared with controls. Collectively, our data suggest that PAX3 functions as an oncogene and plays an important role in BGSCs differentiation by transcriptional regulation of p53.

### p53 Knockdown Promotes the Proliferation and Migration of Differentiated BGSCs

**Figure 6:**
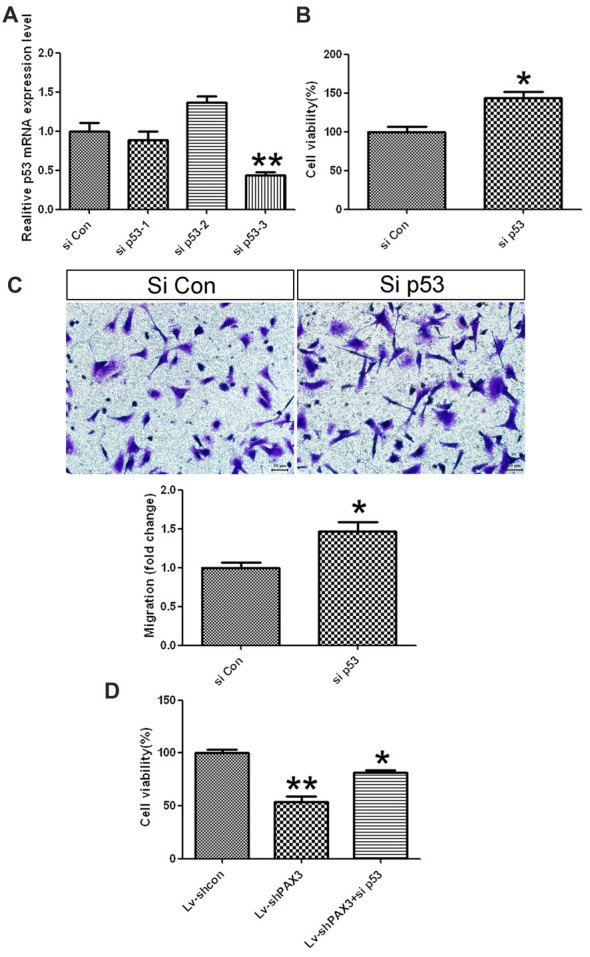
p53 knockdown promotes the proliferation and migration of differentiated BGSCs. **(A)** qRT-PCR showed that the mRNA expressions of p53 in differentiated BGSCs were significantly decreased by knockdown with siRNA-3. **(B)** Knocking down of p53 increased the proliferation of BGSCs. **(C)** si p53 increased the migration of differentiated BGSCs. **(D)** Cell viability assay of BGSCs that had been transfected with Lv-shPAX3 with or without p53 siRNA. p53 silencing attenuated the shPAX3 inhibition on BGSCs proliferation. *p < 0.05 and **p < 0.01 vs. control.

It is well known that p53 can negatively regulate cell growth and migration (Akdemir et al., 2014; Kastenhuber and Lowe, 2017). Hence, we presumed that increased cell proliferation in PAX3 overexpression cells might rely on reduced p53 expression. qRT-PCR results showed that p53 mRNA expression was decreased in differentiated BGSCs transfected with siRNA-3 (Figure 6A). Knocking down of p53 increased the differentiated BGSCs cell viability (Figure 6B) and migration (Figure 6C). Moreover, BGSCs transfected with Lv-shPAX3 and sip53 could partially restored the decrease in cell viability by knocking down PAX3 alone (Figure 6D). This suggested that PAX3 promoted the BGSCs stemness and growth, at least partially, in a p53-dependent manner.

### HIF-1α May Regulate PAX3/p53 Axis Under Hypoxic Conditions

**Figure 7:**
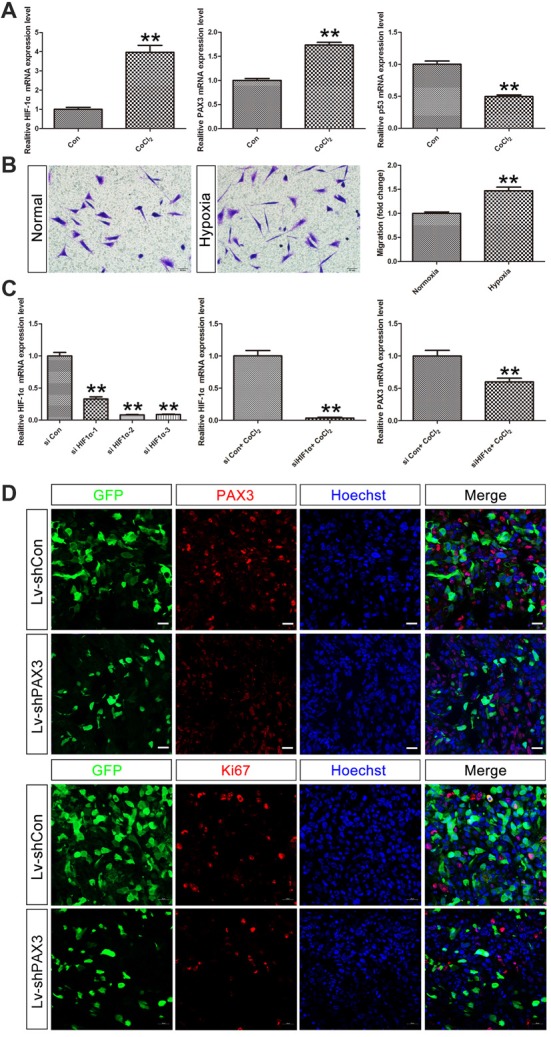
Hypoxia inducible factor-1α (HIF-1α) may regulate PAX3/p53 axis under hypoxic conditions. **(A)** qRT-PCR showed that the mRNA expression of HIF-1α, PAX3 and p53 in differentiated BGSCs that induced hypoxia by CoCl_2_. **(B)** The migration of BGSCs increased under the hypoxic condition. **(C)** Knocking down of HIF-1α reduced the expression of PAX3 in differentiated BGSCs that induced hypoxia by CoCl_2_. **(D)** PAX3 knockdown reduced proliferation of BGSCs *in vivo*. Tumor sections collected from control or PAX3 shRNA-treated groups were processed and subjected to immunofluorescence staining for PAX3 (red) or Ki67 (red). Hoechst 33342 (blue) was used to visualize the nuclei. Scale bars: **(B)**, 50 μm and **(D)** 20 μm. **p < 0.01 vs. negative control (Lv-shCon).

Under hypoxic conditions, HIF-1α, which plays a significant role in stemness maintenance, is steadily expressed (Qiang et al., 2012). Therefore, we determined whether HIF-1α is an upstream regulator of PAX3 and affects the process of “dedifferentiation” under hypoxia induced by CoCl_2_. qRT-PCR results showed that HIF-1α was induced in differentiated BGSCs treated with 500 μmol/L of CoCl_2_ for 48 h (Figure 7A). Moreover, hypoxia induced upregulation of stemness gene PAX3 together with downregulation of p53, indicating that HIF-1α may regulate PAX3/p53 axis under hypoxic conditions. In order to demonstrate the effect of hypoxia on the behavior of BGSCs, we used transwell assay to measure the migration capacity. As shown, hypoxia also significantly promoted the migration potential of BGSCs (Figure 7B). In addition, HIF-1α downregulation reduced the PAX3 expression under hypoxia induced by CoCl_2_, suggesting that HIF-1α is a potential upstream regulator of PAX3 (Figure 7C). Furthermore, PAX3 knockdown reduced the proliferation of BGSCs *in vivo* from tumorigenicity assay (Figure 7D).

**Figure 8:**
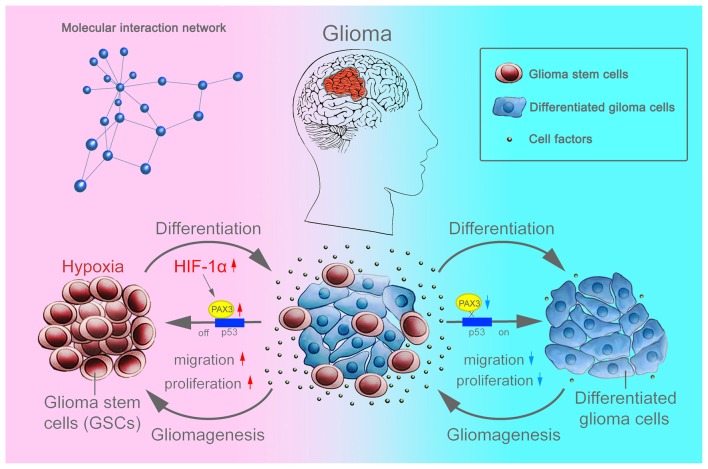
Schematic diagram showing that BGSCs differentiation and gliomagenesis transcriptionally regulated by HIF-1α and PAX3/p53 axis under hypoxic conditions.

Overall, as an important stem cell niche, hypoxic microenvironment promotes the persistence of BGSCs in GBM (Cui et al., 2017). HIF-1α were induced in differentiated BGSCs by hypoxia and then increased the expression of stemness gene PAX3, which further transcriptional repressed the expression of p53, inhibited the differentiation process of BGSCs. In addition, HIF-1α mediated PAX3/p53 axis promoted the process of dedifferentiation under hypoxia, contributing to the cancer stem cell properties, such as proliferation and migration (Figure 8). Knocking down of PAX3 in BGSCs could reverse this process via p53 pathway activation, promoting the BGSCs differentiates to glioma cells.

## Discussion

PAX3 may be a potential oncogene in GBM and is upregulated in tissue specimens of high-grade GBM other than low-grade GBM or normal brain tissues (Chen et al., 2012; Su et al., 2016). Additionally, inactivation of p53 is related to the glioma initiation and progression (Gu et al., 2013). The mutation of p53 was also associated with gliomagenesis. Thus, we further examined whether PAX3 interacts with p53 in human GBM.

Here, we first clarified the correlation between PAX3 and p53 in GBM and glioma cell lines. We compared p53 mutations occurring in PAX3-low vs. PAX3-High glioma samples. More p53 mutations occur in PAX3-high tumors, suggesting a correlation between PAX3 and p53 expression. PAX3-high tumors harbor part of the p53 mutations. Since GBM show notable heterogeneity, both between tumors (inter-tumoral heterogeneity) and within tumors (intra-tumoral and cellular heterogeneity), there is a molecular and cellular complexity and it is unlikely to respond to targeting of single molecular pathways (Jin et al., 2017). Immunofluorescence of p53 was shown activated following BGSCs differentiation. We further identified that PAX3 might specifically bind to the promoter of p53 gene, and transcriptionally repressed p53 expression. ChIP assay further confirmed that PAX3/p53 axis regulated the differentiation process of BGSCs. Then, the function of PAX3 in BGSCs were sequentially investigated *in vitro* and *in vivo*. Ectopic PAX3 expression promoted BGSCs growth and migration while PAX3 knockdown suppresses BGSCs growth, migration *in vitro* and *in vivo*. Similar to PAX3 overexpression, p53 inhibition also showed increase in growth and migration of differentiated BGSCs. Regarding the functional interaction between PAX3 and p53, PAX3 knockdown-mediated decrease in proliferation was partially rescued by p53 inhibition.

Therapeutic targeting of BGSCs by surface markers or signaling pathway, might represent a promising strategy for GBM treatment (Liebelt et al., 2016). Inducing BGSCs differentiation is another approach which is different from targeting the stemness signaling of BGSCs. For example, BMP4 administration could induce BGSCs differentiation and attenuate the formation of BGSCs (Piccirillo et al., 2006). The resveratrol-induced differentiation of BGSCs via the p53/Nanog axis also could be a therapeutic strategy against GBM (Sato et al., 2013). For targeting oligodendroglial-oriented differentiation of BGSCs, another effective approach for anti-gliomagenic treatment was carried out (Wang Y. et al., 2017). p53 pathway also functions in cancer stem cell maintenance and differentiation (Gu et al., 2013; Karsy et al., 2015; Ludwig and Kornblum, 2017). Our results confirmed that p53 activation following the BGSCs differentiation. Additionally, ectopic expression of PAX3 in BGSCs strongly inhibited the expression of p53, suggesting that PAX3-inhibited differentiation is also related to the activity of p53.

Hypoxic microenvironment is also important for the persistence of cancer stem cells (Pietras et al., 2008; Lin and Yun, 2010). HIF-1α was found overexpressed in high grade gliomas (Cohen and Colman, 2015). Another study found that elevation of MGMT expression via HIF-1α in BGSCs contributes to its chemoresistance (Persano et al., 2012). The observation that a hypoxia-driven undifferentiated state contributes to the chemoresistance of GBM compels further effort to define the mechanisms of chemoresistance in BGSCs and look for novel therapeutic approaches to target BGSCs under the hypoxia niche effectively (Li et al., 2009; Pistollato et al., 2010; Seidel et al., 2010). The epigenetic regulation of stemness in brain tumor cells may play a role in this process (Prasad et al., 2017).

In the present work, our hypothesis is that the remaining differentiated cells may be induced dedifferentiation into BGSCs-like cells under hypoxia which play a critical role in the rapid, high-frequency recurrence of GBM. In this dedifferentiation, serum also plays an essential role. The traditional GBM heterogeneity model, cell division model and GBM malignancy development model are challenged by our findings. The mechanism of GBM recurrence and the significance of anti-hypoxia therapy are also highlighted by our present work. Besides BGSCs, resistance of traditional therapy and the rapid, high recurrence of GBM also considerably attribute to remaining differentiated tumor cells.

Both BGSCs and differentiated GBM cells are found to locate in a hypoxic microenvironment (Denko, 2008; Sgubin et al., 2012). The phenotype maintenance, enrichment and tumorigenic capacity of BGSCs are regulated via hypoxia (Sgubin et al., 2012; Li et al., 2013), which plays a crucial role in stemness maintenance (Pistollato et al., 2010). Overall, HIF-1α expresses in hypoxia and promotes the dedifferentiation and vessel formation of GBM cells sequentially. For acquiring the stemness features, the dedifferentiation of differentiated BGSCs is induced by hypoxia (Li et al., 2013). It suggests that oxygen tension should be taken in full concern for development of therapeutic strategies targeting BGSCs (Albert et al., 2014). Whether we should only target BGSCs in the therapy of GBM is another interesting and noteworthy question. Both BGSCs and differentiated GBM cells are suggested to be targeted simultaneously (Wang P. et al., 2017).

In summary, here we revealed several novel tumor characterization and molecular mechanisms: (1) PAX3 expression is elevated in high-grade gliomas and BGSCs derived from GBM; (2) PAX3 is essential for the growth of BGSCs both *in vitro* and *in vivo*; (3) PAX3 and p53 expression in BGSCs is mutually-exclusive and PAX3 negatively regulates p53 action on BGSCs; (4) PAX3 transcriptionally represses p53 expression in BGSCs and differentiation; (5) hypoxia increases the expression of HIF-1α and PAX3 in differentiated BGSCs while reduces p53 expression. Therefore, HIF-1α mediated PAX3/p53 axis is an essential mechanism that controls self-renewal, migration, and tumorigenesis of BGSCs in a p53-dependent manner.

## Author Contributions

CX and QHan conceived and designed the experiments. HZ, HW and QHuang carried out the experiments. QL, YG and JL analyzed the data. XL and CX contributed reagents/materials. HZ and CX prepared and revised the manuscript. The final manuscript has been read and approved by all the authors.

## Conflict of Interest Statement

The authors declare that the research was conducted in the absence of any commercial or financial relationships that could be construed as a potential conflict of interest. The reviewer VR and handling Editor declared their shared affiliation.
